# Gray matter asymmetry atypical patterns in subgrouping minors with autism based on core symptoms

**DOI:** 10.3389/fnins.2022.1077908

**Published:** 2023-01-25

**Authors:** Cuicui Li, Wenxiong Chen, Xiaojing Li, Tong Li, Ying Chen, Chunling Zhang, Mingmin Ning, Ximing Wang

**Affiliations:** ^1^Shandong Provincial Hospital Affiliated to Shandong First Medical University, Jinan, China; ^2^Guangzhou Women and Children’s Medical Center, Guangzhou, China; ^3^Central Hospital Affiliated to Shandong First Medical University, Jinan, China

**Keywords:** autism, gray matter asymmetry, minors, core symptom domains, brain-behavior relationships

## Abstract

Abnormal gray matter (GM) asymmetry has been verified in autism spectrum disorder (ASD), which is characterized by high heterogeneity. ASD is distinguished by three core symptom domains. Previous neuroimaging studies have offered support for divergent neural substrates of different core symptom domains in ASD. However, no previous study has explored GM asymmetry alterations underlying different core symptom domains. This study sought to clarify atypical GM asymmetry patterns underlying three core symptom domains in ASD with a large sample of 230 minors with ASD (ages 7–18 years) and 274 matched TD controls from the Autism Brain Imaging Data Exchange I (ABIDE I) repository. To this end, the scores of the revised autism diagnostic interview (ADI-R) subscales were normalized for grouping ASD into three core-symptom-defined subgroups: social interaction (SI), verbal communication (VA), and restricted repetitive behaviors (RRB). We investigated core-symptom-related GM asymmetry alterations in ASD resulting from advanced voxel-based morphometry (VBM) by general linear models. We also examined the relationship between GM asymmetry and age and between GM asymmetry and symptom severity assessed by the Autism Diagnostic Observation Schedule (ADOS). We found unique GM asymmetry alterations underlying three core-symptom-defined subgroups in ASD: more rightward asymmetry in the thalamus for SI, less rightward asymmetry in the superior temporal gyrus, anterior cingulate and caudate for VA, and less rightward asymmetry in the middle and inferior frontal gyrus for RRB. Furthermore, the asymmetry indexes in the thalamus were negatively associated with ADOS_SOCIAL scores in the general ASD group. We also showed significant correlations between GM asymmetry and age in ASD and TD individuals. Our results support the theory that each core symptom domain of ASD may have independent etiological and neurobiological underpinnings, which is essential for the interpretation of heterogeneity and the future diagnosis and treatment of ASD.

## 1. Introduction

Autism spectrum disorder (ASD) encompasses a group of pervasive neurodevelopmental disorders characterized by three core symptom domains: social interaction deficits (SI), verbal communication abnormalities (VA), and restricted repetitive behaviors (RRB) (Shulman et al., 2020). All three core symptom domains encompass various behaviors that manifest diversely across the disease, making ASD a condition with complex behaviors. While ASD is a common neurodevelopmental condition and has been investigated in numerous studies, it is also remarkably heterogeneous, complicating its diagnosis and neuroimaging findings. Over the past 30 years, the definition of autism has undergone a constant transformation, leading to the current autism spectrum (Shulman et al., 2020).

Identifying neuroimaging is vital to understanding the etiology and pathophysiology of ASD patients. Such markers can lead to early diagnosis and better treatment (Loth et al., 2016), particularly early in life, when interventions can have the greatest effect on patients with ASD. Although the exact neuroimaging changes in patients with ASD remain unknown, some brain structural alterations seem to be involved. These changes include decreased cortical thickness in the right pre- and postcentral gyrus and increased cortical thickness in the superior temporal sulcus, cingulate gyrus, and fusiform gyrus (Hardan et al., 2006; Hyde et al., 2010); greater gray matter (GM) volume in the amygdala, bilateral superior temporal gyrus, and precuneus and lower GM volume in the right inferior temporal gyrus in children with ASD (Retico et al., 2016; Lucibello et al., 2019); atypical cortical thickness development with accelerated expansion followed by accelerated thinning and atypical cortical volume development with increased cortical total volume (especially in the frontal and temporal lobes) in young ASD children (Li et al., 2017; Rommelse et al., 2017); and altered brain structure asymmetry with significantly increased rightward asymmetry in the posterior superior temporal gyrus, inferior parietal lobule, and auditory cortex and reduced rightward asymmetry in the parahippocampal gyrus, fusiform- and inferior temporal thickness (Gage et al., 2009; Floris et al., 2016; Postema et al., 2019).

Brain asymmetry is thought to be an evolutionary adaptation ensuring more efficient transcortical information integration and avoiding redundancy in cognitive processing (Hugdahl, 2011). Alterations in brain asymmetry have been shown to influence a variety of neurological and psychotic disorders, especially ASD (Prior and Bradshaw, 1979). ASD patients exhibit deficits in left hemisphere skills, such as language and motor skills, while right hemisphere skills remain relatively unaffected (McCann, 1982). This pattern in ASD has spawned theories that attempt to reconcile its complex clinical features with atypical brain asymmetry. However, there are mixed findings on brain structural asymmetry alterations in patients with ASD (Postema et al., 2019). For instance, a study showed increased leftward asymmetry of GM volume in the language-association areas in ASD (Hazlett et al., 2006). However, research by De Fossé et al. (2004) found rightward asymmetry of GM volume in the language-related cortex in ASD.

Untangling the heterogeneity of brain structural asymmetry may lead to the improvement of accurate diagnoses and elaborate clinical subgrouping and may help in developing targeted treatment plans for patients with ASD. Regarding heterogeneity, most studies focus on ex-factors, such as age, comorbidities, medication use, and methodological differences (Hobson and Petty, 2021). However, Haar et al. (2016) found that differences between the ASD group and the control group were overshadowed by considerable within-group variability. Happé et al. (2006) demonstrated that each core symptom domain of ASD has independent etiological and neurobiological underpinnings. Ronald et al. (2006) studied the genetic etiology of autistic traits in 3,419 normal child twin pairs and found that unique genetic variance was associated with each of the three core symptom domains. As such, individuals will vary within a huge permutation network, unavoidably leading to a highly heterogeneous population. Additionally, previous neuroimaging studies of ASD have offered support for the divergent neural substrates of different core symptom domains, which are mediated by partially distinct brain regions. For instance, social cognition mainly relies on certain social brain regions, such as the fusiform face area, amygdala, medial prefrontal cortex, anterior cingulate cortex, superior temporal sulcus, and inferior frontal gyrus, RRB has been linked to abnormalities in motor regions, and reduced activation in the right parahippocampal gyrus, cerebellum, left anterior cingulate, and bilateral cingulate in face-processing social tasks has been reported in patients with ASD (Spencer et al., 2011; Patriquin et al., 2016; Duret et al., 2018; Wilkes and Lewis, 2018). In addition, our previous research showed that atypical gyrification patterns encode changes in the symptom dimensions of ASD (Ning et al., 2021). The analysis is complicated by likely distinct symptom clusters or subgroups that exist among patients with ASD (Lenroot and Yeung, 2013). Significant intra- and interindividual variabilities in patients with ASD make it challenging to reliably determine the neural mechanisms of the disorder. Therefore, it is necessary to subcategorize patients with ASD according to core symptoms to explore their brain structural asymmetry. However, no previous study thus far has explored this issue.

Based on the above research, we hypothesize that subgroups dominated by different core symptom domains may have unique brain structural asymmetry alterations because each subgroup has differential clinical manifestations and neurobiological underpinnings. However, we also assumed that there would be shared brain structural asymmetry alterations because these subgroups are on the same disease spectrum and have partly similar pathological mechanisms. For this, an advanced voxel-based morphometry (VBM) method established by Kurth et al. (2015) and a large sample (230 minors with ASD, 274 control subjects) from the Autism Brain Imaging Data Exchange I (ABIDE I) repository was used to clarify GM asymmetry atypical patterns underlying three core symptom domains and to further explore the relationship between GM asymmetry and age and between abnormal GM asymmetry and symptom severity measured by the Autism Diagnostic Observation Schedule (ADOS).

## 2. Participants and methods

### 2.1. Participant recruitment

A total of 230 minors with ASD (ages 7–18 years) and 274 matched typically developing (TD) controls were examined from ABIDE I: a consortium with 539 individuals with ASD and 573 TD controls (ages 7–64 years) collected from 17 sites. Our data were aggregated across right sites that included at least five individuals with ASD and five age-, gender-, and site-matched TD controls who met our inclusion criteria. Briefly, we selected individuals aged 7–18 (the ages most represented in ABIDE I sites) with good quality T1-weighted images that can be preprocessed successfully, with complete revised autism diagnostic interview (ADI-R) scores.

To investigate whether GM asymmetry differs across ASD dominated by different core symptom domains, we divided minors with ASD into three subgroups, SI, VA, and RRB based on the ADI-R (Bokadia et al., 2020). The ADI-R is a semistructured scale that evaluates and scores the condition mainly through interviews with patients’ parents, including the ADI-R-SOCIAL-TOTAL-A (corresponding to SI), ADI-R-VERBAL-TOTAL-BV (corresponding to VA), and ADI-R-RRB-TOTAL-C (corresponding to RRB) subscales. First, we used the corresponding maximal values to normalize the scores of three ADI-R subscales according to the following formula and then obtained three values between 0 and 1, denoted as S, V, and R, respectively (Bokadia et al., 2020).


$${\text{ X}}^{\prime }\text{=}\frac{\text{X}}{\text{X\_M A X}}\;$$

Finally, minors with ASD were assigned to three subgroups according to the normalized scores: subjects with S as the largest value were included in the SI subgroup; subjects with V as the largest value were included in the VA subgroup; subjects with R as the largest value were included in the RRB subgroup. If S = V or S = R or V = R, he would be excluded. TD controls were randomly selected to form age-, gender- and site-matched TD groups. To maximize the sample size, each TD control was matched four times, meaning that some controls may have appeared in multiple subgroups. Ultimately, 230,146, 43, and 41 patients were included in the general ASD, SI, VA, and RRB subgroups, respectively, and 264, 249, 127, and 111 controls were included in the corresponding TD subgroups.

The ADI-R was from the parents’ assessment, which lacked an assessor’s objective evaluation of clinical symptoms. To prevent any influence of subjective parents’ assessment on the subgroup, we conducted subgroups based on ADOS with the same methodology. ADOS is a semistructured standard diagnostic tool employing a play-based approach to detect autistic symptoms. Ultimately, 148,19, and 0 patients were included in the SI_2_, VA_2_, and RRB_2_ subgroups based on ADOS, respectively, and 225, 55, and 0 controls were included in the corresponding TD subgroups.

All data were anonymized and collected by studies approved by the regional Institutional Review Boards.

### 2.2. MRI data acquisition

Whole-brain high-resolution T1-weighted anatomical images of all participants in our study were acquired in ABIDE I, and detailed parameters and acquisition protocols used at each site can be seen at http://fcon_1000.projects.nitrc.org/indi/abide/.

### 2.3. Image analysis

#### 2.3.1. Image preprocessing

In our study, we preprocessed 3D T1-weighted images using VBM^1^ toolbox for Statistical Parametric Mapping (SPM8^2^) software. Before preprocessing, visual checks were performed for all images. (1) Segment tissue: the symmetrical tissue probability map downloaded from the internet^3^ was used to perform tissue segmentation on 3D T1-weighted images to obtain GM, white matter (WM) and cerebrospinal fluid segments, and quality inspection was performed. (2) Flip tissue segments: the GM and WM segments obtained in the last step were flipped with the midline of the brain as the axis to create new images with reversed left and right brain hemispheres. (3) Generate a symmetrical template: the flipped images obtained in step 2 and the original versions obtained in step 1 were used to generate a symmetrical Diffeomorphic Anatomical Registration Lie (DARTEL) template. (4) Warp images: unflipped and flipped GM images obtained in steps 1 and 2, respectively, were warped to the symmetric DARTEL template obtained in step 3. (5) Generate the right-hemisphere mask: a right-hemisphere mask was generated in MRIcron^4^ using the DARTEL template obtained in step 3 to limit further analysis to the right hemisphere.

#### 2.3.2. Estimation of the asymmetry index (AI)

All warped original GM versions and their corresponding warped flipped GM segments (both generated in step 4) and the right-hemispheric mask (obtained in step 5) were selected to calculate the voxel-wise GM asymmetry following the optimized protocol by Kurth et al. (2015). In this step, the left hemispheres were discarded for all original and flipped GM versions of all subjects during the masking procedure. As a result, the original and flipped GM segments yielded the right and left hemispheres, respectively (Kurth et al., 2015). Then, the masked asymmetry index (AI) images of each participant were generated according to the following formula: AI = ((i1–i2)/((i1+i2).*0.5)).*i3, in which i1 and i2 are original and flipped GM warped images, respectively, and i3 is the right-hemispheric mask image (Kurth et al., 2015). The resulting AI images were then spatially smoothed with an 8 mm Gaussian kernel. In the results, positive and negative AI values indicate rightward and leftward asymmetry, respectively.

### 2.4. Statistical analyses

Differences in gender between each subgroup pair were tested using the chi-square test in SPSS software (IBM Corp, Armonk, NY), and other variables were tested using the independent two-sample *t*-test. At an uncorrected threshold of *P* < 0.05, the results were considered statistically significant. For demographic differences among the three subgroups, the nonparametric test was used for ADI-R subscores, and *P* < 0.05 was considered statistically significant after Bonferroni correction by SPSS software.

To explore which brain regions exhibited differences in GM asymmetry between the ASD/SA/VI/RRB group and corresponding TD groups, general linear models were evaluated, with age, gender, and site as covariates in SPM8. When the participants belong to site 1, site 1 was recorded as 1 and other sites were recorded as 0 (other sites = 0, site 1 = 1). The same was true for the other sites. In the Data Preprocessing Assistant for rs-fMRI software (DPARSF^5^; Yan et al., 2016), brain clusters with significant differences in between-group comparisons were saved as masks.

In addition, to test the relationship between symptom severity measured by ADOS subscores and AIs in the general ASD group, multiple regression analyses were implemented, with age, gender, and site as covariates in SPM8. Scatter plots describing the linear relation between the clinical severity and AIs were conducted in SPSS. Because few ASD participants were using ADOS in three subgroups based on ADI-R, we performed regression analyses only in the general ASD group; ultimately, 129 ASD patients in the general ASD group were used for regression analyses. Similarly, to explore the relationship between AIs and age in patients with ASD and TD controls, multiple regression analyses were implemented, with gender and site as covariates. Scatter plots describing the linear relation between age and AIs were generated in SPSS. Importantly, multiple regression analyses concentrated on masks saved in the above between-group comparisons.

In all general linear models and multiple regression analyses, the results were corrected for multiple comparisons using the Gaussian random field (GRF) procedure with the voxel level of *P*-value < 0.005 and the cluster level of *P* < 0.05 implemented by DPARSF (Deng and Wang, 2021).

## 3. Results

### 3.1. Demographic and clinical details

Descriptive statistics are presented in Tables 1, 2 and Supplementary Table 1. There were no significant differences between each subgroup pair in age and gender (*P* > 0.05). Three subgroups significantly differed in ADI-R subscale scores (*P* < 0.05).

**TABLE 1:** Participant demographics.

	Variables		NYU	UCLA	UM	KKI	PITT	STANFORD	TRINITY	YALE	Total
ASD and TD	Subjects (N)	ASD (TD)	56 (65)	52 (45)	65 (62)	14 (25)	7 (9)	14 (20)	9 (14)	13 (24)	230 (264)
	Age (years)	ASD	11.23 ± 2.62	13.04 ± 2.50	13.00 ± 2.34	10.26 ± 1.46	12.80 ± 1.00	9.86 ± 1.63	14.62 ± 1.75	12.45 ± 2.97	12.25 ± 2.61
		TD	12.06 ± 2.79	12.96 ± 1.92	13.69 ± 2.59	10.37 ± 1.31	13.32 ± 1.00	9.95 ± 1.60	14.33 ± 1.63	12.52 ± 2.57	12.48 ± 2.58
	Gender M (F)	ASD	56 (10)	47 (5)	55 (10)	13 (1)	4 (3)	11 (3)	9 (0)	10 (3)	199 (31)
		TD	50 (13)	39 (6)	50 (12)	24 (1)	7 (2)	16 (4)	14 (0)	17 (7)	219 (45)
	Statistics	Age	*t* = −1.69, *p* = 0.09	*t* = 0.33, *p* = 0.57	*t* = −1.56, *p* = 0.12	*t* = −0.25, *p* = 0.80	*t* = −1.03, *p* = 0.32	*t* = −0.12, *p* = 0.90	*t* = 0.41, *p* = 0.69	*t* = −0.73, *p* = 0.94	*t* = −1.00, *p* = 0.32
		Gender	*X ^2^* = 0.40, *p* = 0.53	*X ^2^* = 0.17, *p* = 0.87	*X ^2^* = 0.35, *p* = 0.56	*X ^2^* = 0.18, *p* = 0.67	*X ^2^* = 0.78, *p* = 0.38	*X ^2^* = 0.01, *p* = 0.92	–	*X ^2^* = 0.16, *p* = 0.69	*X ^2^* = 1.20, *p* = 0.27
SI and TD	Subjects (N)	SI (TD)	30 (58)	16 (34)	16 (44)	14 (25)	7 (9)	14 (20)	9 (14)	13 (24)	146 (249)
	Age (years)	SI	11.69 ± 2.67	12.80 ± 2.55	12.68 ± 2.30	10.26 ± 1.46	12.80 ± 1.00	9.89 ± 1.63	14.62 ± 1.75	12.45 ± 2.97	12.25 ± 2.56
		TD	12.59 ± 2.82	13.03 ± 1.73	13.19 ± 2.46	10.37 ± 1.31	13.31 ± 1.00	9.95 ± 1.60	14.33 ± 1.63	12.52 ± 2.57	12.49 ± 2.47
	Gender M (F)	SI	28 (2)	15 (1)	15 (1)	13 (1)	4 (3)	11 (3)	9 (0)	10 (3)	124 (22)
		TD	54 (4)	29 (5)	43 (1)	24 (1)	7 (2)	16 (4)	14 (0)	17 (7)	213 (36)
	Statistics	Age	*t* = −1.44, *p* = 0.15	*t* = −0.37, *p* = 0.71	*t* = −0.72, *p* = 0.48	*t* = −0.25, *p* = 0.80	*t* = −1.03, *p* = 0.32	*t* = −0.12, *p* = 0.90	*t* = 0.41, *p* = 0.69	*t* = −0.93, *p* = 0.36	*t* = −0.49, *p* = 0.62
		Gender	*X ^2^* = 0.002, *p* = 0.97	*X ^2^* = 0.74, *p* = 0.39	*X ^2^* = 0.58, *p* = 0.45	*X ^2^* = 0.18, *p* = 0.67	*X ^2^* = 0.78, *p* = 0.38	*X ^2^* = 0.01, *p* = 0.92	–	*X ^2^* = 0.16, *p* = 0.69	*X ^2^* = 0.03, *p* = 0.87
	Variables		NYU	UCLA	UM	Total
RRB and TD	Subjects (N)	RRB (TD)	10 (33)	15 (34)	16 (44)	41 (111)
	Age (years)	RRB	11.14 ± 2.59	12.70 ± 2.60	12.45 ± 2.13	12.22 ± 2.45
		TD	11.90 ± 2.06	13.02 ± 1.73	13.19 ± 2.46	12.75 ± 2.19
	Gender M (F)	RRB	10 (0)	14 (1)	15 (1)	39 (2)
		TD	33 (0)	29 (5)	43 (1)	106 (5)
	Statistics	Age	*t* = −0.96, *p* = 0.34	*t* = −0.49, *p* = 0.62	*t* = −1.06, *p* = 0.29	*t* = −1.28, *p* = 0.20
		Gender	–	*X ^2^* = 0.30, *p* = 0.59	*X ^2^* = 0.58, *p* = 0.45	*X ^2^* = 0.01, *p* = 0.92
VA and TD	Subjects (N)	VA (TD)	16 (48)	10 (35)	17 (44)	43 (127)
	Age (years)	VA	10.42 ± 2.47	13.00 ± 2.75	13.42 ± 2.73	12.21 ± 2.93
		TD	11.16 ± 2.40	13.00 ± 2.10	14.66 ± 2.67	12.88 ± 2.83
	Gender M (F)	VA	12 (4)	8 (2)	16 (1)	36 (7)
		TD	33 (15)	31 (4)	43 (1)	107 (20)
	Statistics	Age	*t* = −1.06, *p* = 0.29	*t* = 0.00, *p* = 1	*t* = −1.60, *p* = 0.12	*t* = −1.34, *p* = 0.18
		Gender	*X ^2^* = 0.23, *p* = 0.64	*X ^2^* = 0.50, *p* = 0.48	*X ^2^* = 0.50, *p* = 0.48	*X ^2^* = 0.07, *p* = 0.93

ASD, autism spectrum disorder; TD, typically developing; SI, subgroup dominated by social interaction deficits; VA, subgroup dominated by verbal communication abnormalities; RRB, subgroup dominated by restricted repetitive behaviors; KKI, Kennedy Krieger Institute; NYU, New York University Langone Medical Center; UCLA, University of California-Los Angeles; UM, University of Michigan; YALE, Yale Child Study Center; STANFORD: Stanford University; TRINITY, Trinity Centre for Health Sciences; PITT, University of Pittsburgh School of Medicine.

**TABLE 2:** Differences in the three ADI-R subscales among the three ASD subgroups.

Variables	Std. Test Statistic	*P*-value*	Std. Test Statistic	*P*-value*	Std. Test Statistic	*P*-value*
	SI vs. VA		SI vs. RRB		VA vs. RRB	
ADI-R-SOCIAL	−4.295	< 0.0001	3.727	< 0.0001	0.607	1.000
ADI-R-VERBAL	−2.666	0.023	0.512	1.000	−4.759	< 0.0001
ADI-R-RRB	−0.524	1.000	6.909	< 0.0001	5.178	< 0.0001

ADI-R-SOCIAL, social subscore of autism diagnostic interview-revised (ADI-R); ADI-R-VERBAL, verbal subscore of ADI-R; ADI-R-RRB, restricted repetitive behaviors subscore of ADI-R; SI, subgroup dominated by social interaction deficits; VA, subgroup dominated by verbal communication abnormalities; RRB, subgroup dominated by restricted repetitive behaviors.

*Bonferroni corrected p-values.

### 3.2. Differences in GM asymmetry between general ASD and TD controls

**FIGURE 1:**
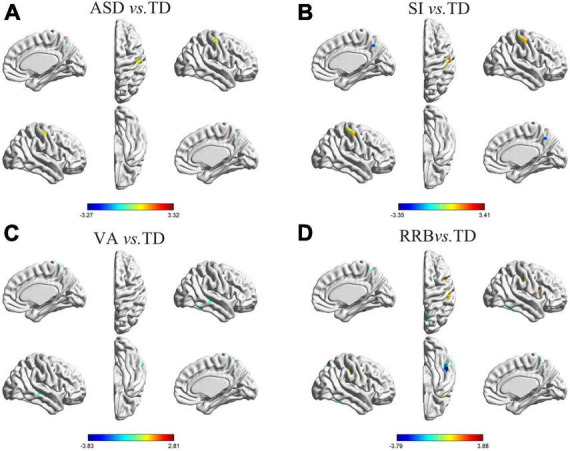
Differences in GM asymmetry in between-group comparisons. **(A)** General ASD vs. TD controls: seven clusters with significant differences between general ASD and TD controls. **(B)** SI vs. TD: four clusters with significant differences between SI and TD controls. **(C)** VA vs. TD: four clusters with significant differences between VA and TD controls. **(D)** RRB vs. TD: five clusters with significant differences between RRB and TD controls; the red color indicates the general ASD/SI/VA/RRB group with more rightward asymmetry, and the blue color indicates more leftward asymmetry. The results were corrected for multiple comparisons using the Gaussian random field procedure with the voxel level *P*-value < 0.005 and the cluster level of *P* < 0.05. GM, gray matter; ASD, autism spectrum disorder; TD, typically developing; SI, subgroup dominated by social interaction deficits; VA, subgroup dominated by verbal communication abnormalities; RRB, subgroup dominated by restricted repetitive behaviors.

As shown in Figure 1A and Table 3, specific brain regions showed significant differences between general ASD and TD controls: ASD patients had more leftward asymmetry in Cluster A1 (inferior temporal/middle temporal gyrus), Cluster A2 (parahippocampal gyrus), Cluster A3 (superior temporal gyrus), and Cluster A6 (precuneus) and had more rightward asymmetry in Cluster A4 (thalamus), Cluster A5 (medial frontal gyrus) and Cluster A7 (postcentral/precentral gyrus).

**TABLE 3:** Coordinates of clusters with significant differences in between-group comparisons.

	Cluster	Location	MNI coordinates			voxels	*t*-value
			**x**	**y**	**z**		
ASD vs. TD	A1	Inferior Temporal Gyrus/Middle Temporal Gyrus	67.5	−34.5	−22.5	268/126	-3.271
	A2	ParaHippocampal	13.5	1.5	−24	69	-3.170
	A3	Superior Temporal Gyrus	42	10.5	−16.5	41	-2.810
	A4	Thalamus	19.5	−18	12	127	3.169
	A5	Medial Frontal Gyrus	12	37.5	25.5	5	2.739
	A6	Precuneus	3	−60	45	106	-2.998
	A7	Postcentral Gyrus/Precentral Gyrus	55.5	−24	60	233/145	3.053
SI vs. TD	S1	Middle Temporal Gyrus	60	−24	−9	67	-2.986
	S2	Thalamus	19.5	−18	12	65	2.955
	S3	Precuneus	3	−58.5	46.5	182	-3.355
	S4	Postcentral/Precentral Gyrus	55.5	−25.5	60	456/172	3.411
VA vs. TD	V1	Inferior Temporal Gyrus	66	−48	−19.5	78	-3.829
	V2	Middle Temporal/Superior Temporal Gyrus	45	−27	−1.5	133/102	-3.310
	V3	Anterior Cingulate/Caudate	9	22.5	−3	75/56	-3.055
	V4	Precuneus/Postcentral	9	−58.5	67.5	146/75	-2.973
RRB vs. TD	R1	Middle Temporal Gyrus	57	6	−24	42	-2.678
	R2	Inferior Frontal/Middle Frontal Gyrus	25.5	31.5	−25.5	81/60	-2.869
	R3	Inferior Temporal Gyrus/Fusiform	48	−40.5	−25.5	607/245	-3.794
	R4	Postcentral/Precentral	48	9	22.5	536/204	3.877
	R5	Precuneus	16.5	−72	57	103	-3.017

ASD, autism spectrum disorder; TD, typically developing; SI, subgroup dominated by social interaction deficits; VA, subgroup dominated by verbal communication abnormalities; RRB, subgroup dominated by restricted repetitive behaviors.

### 3.3. Differences in GM asymmetry between ASD subgroups and TD controls

Differences in GM asymmetry between the SI/VA/RRB group and corresponding TD groups were then assessed (Figures 1B–D and Table 3). In particular, relative to the TD group, the SI subgroup showed significantly more rightward asymmetry in Cluster S2 (thalamus) and Cluster S4 (postcentral/precentral gyrus) and less rightward asymmetry in Cluster S1 (middle temporal gyrus) and Cluster S3 (precuneus). The VA subgroup showed significantly less rightward asymmetry than the TD group in Cluster V1 (inferior temporal gyrus), Cluster V2 (middle temporal/superior temporal gyrus), Cluster V3 (anterior cingulate/caudate), and Cluster V4 (precuneus/postcentral gyrus). Relative to the TD group, the RRB subgroup showed significantly more rightward asymmetry in Cluster R4 (precentral/postcentral gyrus) and less rightward asymmetry in Cluster R1 (middle temporal gyrus), Cluster R2 (inferior frontal/middle frontal gyrus), Cluster R3 (inferior temporal/fusiform gyrus), and Cluster R5 (precuneus).

### 3.4. GM asymmetry results validation

GM asymmetry results between ADOS-based ASD subgroups and TD controls overlapped in main brain areas with GM asymmetry findings based on ADI-R, suggesting that our subgrouping based on ADI-R was robust (Supplementary Figure 1 and Supplementary Table 2).

### 3.5. Brain-behavior relationships

**FIGURE 2:**
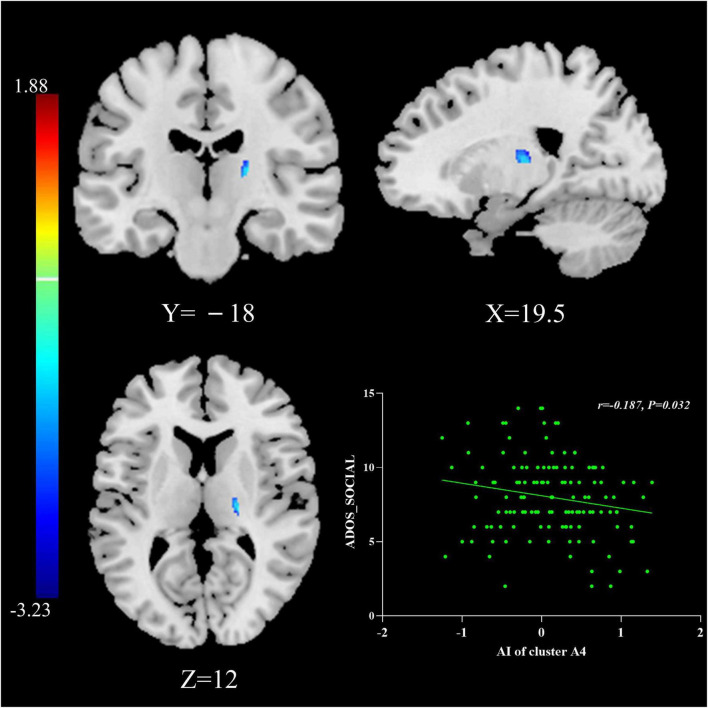
The relationship between AIs and symptom severity. AIs of Cluster A4 were negatively associated with social scores measured by the ADOS in the general ASD group. The results were corrected for multiple comparisons using the Gaussian random field procedure with the voxel level *P*-value < 0.005 and the cluster level of *P* < 0.05. ASD, autism spectrum disorder; AI, asymmetry index; ADOS, Autism Diagnostic Observation Schedule.

The multiple regression analyses using clinical severity measured by ADOS as the independent predictor of AI showed significant negative associations: AIs of Cluster A4 were negatively associated with social scores measured by the ADOS in the general ASD group (*r* = −0.187, *P* = 0.032; Figure 2).

### 3.6. The effect of age

**FIGURE 3:**
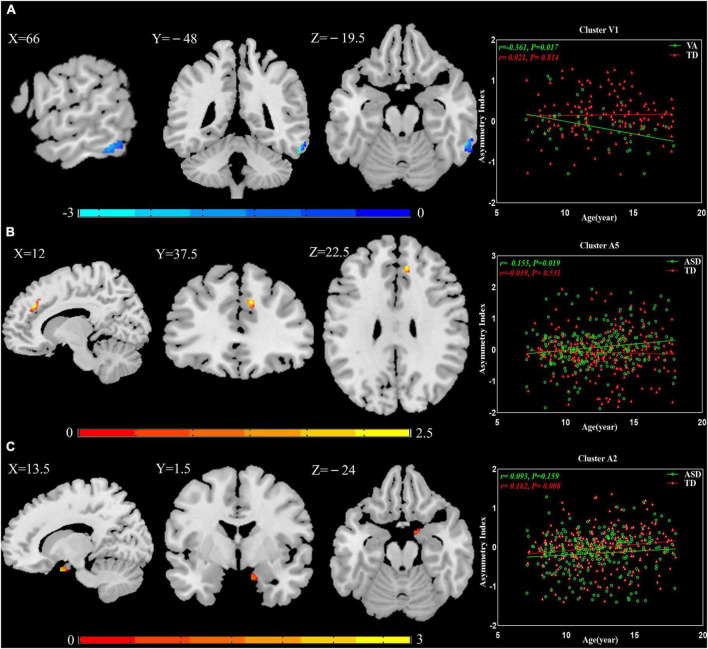
The relationship between AIs and age. **(A)** Cluster V1 exhibited increased leftward asymmetry from age 7 to 18 in the VA subgroup. **(B)** Cluster A5 presented increased rightward asymmetry with age in the general ASD group. **(C)** Cluster A2 showed increased rightward asymmetry over time in the TD group. The results were corrected for multiple comparisons using the Gaussian random field procedure with the voxel level *P*-value < 0.005 and the cluster level of *P* < 0.05. AI, asymmetry index; ASD, autism spectrum disorder; TD, typically developing; VA, subgroup dominated by verbal communication abnormalities.

Multiple regression analyses were calculated between AIs and age in patients with ASD and TD controls: Cluster V1 exhibited increased leftward asymmetry from age 7 to 18 in the VA subgroup (*r* = −0.361, *P* = 0.017), Cluster A5 presented increased rightward asymmetry with age in the general ASD group (*r* = 0.155, *P* = 0.019), and Cluster A2 showed increased rightward asymmetry over time in the TD group (*r* = 0.162, *P* = 0.008; Figure 3).

## 4. Discussion

In this study, we attempted to explore atypical GM asymmetry patterns and subcategorize the neuroimaging of ASD based on its core symptoms in a large brain imaging database (ABIDE I). We found unique and elaborate GM asymmetry patterns among the three ASD subgroups.

### 4.1. The atypical pattern of GM asymmetry in the general ASD group

Abnormal structural asymmetries in patients with ASD have been reported to be widely distributed across various brain regions (Maximo et al., 2014). Examples include significantly increased rightward asymmetry in the inferior parietal lobule, auditory cortex (Floris et al., 2016), posterior superior temporal gyrus (Gage et al., 2009), and lateral orbitofrontal surface area (Postema et al., 2019) and reduced rightward asymmetry in the medial orbitofrontal surface area, putamen volume, and the parahippocampal gyrus (Postema et al., 2019; Li et al., 2021). The reported multiregional asymmetry alterations are consistent with the notion that laterality is easily disrupted in patients with ASD (Maximo et al., 2014). In our study, the results showed that various GM asymmetry alterations were located in several clusters in the general ASD group, predominantly involving the temporal, frontal, and parahippocampal gyrus, which part overlapped with findings in previous studies, such as the parahippocampal gyrus having more leftward asymmetry (Li et al., 2021). However, in the postcentral gyrus, general ASD showed more rightward asymmetry; previous studies by Hau et al. (2022) reported leftward asymmetry of the postcentral gyrus volume in ASD patients relative to the TD group. Interestingly, we found that this brain region exhibited more rightward asymmetry in the SI and RRB subgroups but leftward asymmetry in the VA subgroup. Furthermore, our analysis revealed additional GM asymmetry alterations, including the anterior cingulate, caudate, inferior frontal gyrus, middle frontal gyrus, and fusiform, that were present only in ASD subgroups but not in the general ASD group. Our results further suggested the neuroimaging heterogeneity of ASD. Such heterogeneity would result in the truth being offset or inaccurate if ASD is considered as a whole.

### 4.2. Divergent GM asymmetry patterns in ASD subgroups

Different GM asymmetry alterations were presented across three core-symptom-defined ASD subgroups in our study, consistent with previous notions that brain structure and function alterations are associated with specific clinical symptoms in ASD. For instance, social cognition mainly relies upon certain social brain regions (Patriquin et al., 2016). The results of our study indicated that different clinical phenotypes defined by divergent core-symptom domains would result in heterogeneity in neuroimaging.

Specifically, there was more rightward asymmetry in the thalamus for SI, less rightward asymmetry in the superior temporal gyrus, anterior cingulate, and caudate for VA, and less rightward asymmetry in the middle and inferior frontal gyrus for RRB.

The thalamus, known as the subcortical-cortical relay, is related to social deficits and is thought to be a vital region in ASD (Schuetze et al., 2016). Although MRI cannot provide cellular-level evidence for changed thalamic volume asymmetry, alterations in thalamic volume may be concerned with changes in myelination or other properties of WM axons innervating the region or changes in dendritic or neuropil compartments (Schuetze et al., 2016). Structurally, the rightward asymmetry of thalamic volume modulated the correlation between parental alienation and the social abilities of children with social anxiety disorder (Zhang et al., 2020). On the functional level, hypoconnectivity between the posterior thalamus and parieto-occipital cortex has been found in ASD (Nair et al., 2013), and the pulvinar nucleus has been shown to have significant connections with the prefrontal and parieto-occipital cortices in studies on humans (Arcaro et al., 2015). The occipital abnormalities have been related to social reciprocity deficits in ASD (Amaral et al., 2008). Our results indicate that thalamic volume properties at the structural level may result in alterations in these functional connectivities, which, in turn, influence social interaction in ASD.

The superior temporal gyrus (STG) is one of the language-functional regions in normally developed and language-impaired subjects. During embryologic and early postnatal development, the right STG is equally capable of language processing as the left STG (Kertesz et al., 1992). Nevertheless, this equivalent capacity starts to vary during later development, with the left STG showing a clear advantage in language processing (Kertesz et al., 1992). Reports on language-association area asymmetry in ASD have had inconsistent results, with research showing increased leftward asymmetry of fusiform- and inferior temporal thickness (Postema et al., 2019), study reporting increased leftward asymmetry of GM volume in the language-association areas (Hazlett et al., 2006), lack of planum temporale leftward asymmetry (Rojas et al., 2005), or investigation exhibiting rightward asymmetry of GM volume in language-related cortex in ASD (De Fossé et al., 2004). The fact that patients in the VA subgroup in our study exhibited less rightward asymmetry again provides evidence for atypical GM asymmetry in ASD, albeit admittedly in a different pattern than much of the prior literature.

The frontostriatal system plays a key role in social motivation, which is thought to be the basis of abnormities in verbal communication in ASD (Delmonte et al., 2013). A previous study showed that the mechanisms controlling the number and volume of neurons and the total volume of the caudate nucleus are dysregulated in ASD (Hollander et al., 2005). These cellular alterations result in different volumes of the bilateral caudate (i.e., abnormal caudate asymmetry) in ASD. Abnormal caudate asymmetry underlies the disruption of structural and functional connectivities between the frontal cortex (including anterior cingulate) and striatum (Delmonte et al., 2013), which may potentially explain the link between the leftward asymmetry of the caudate and anterior cingulate and verbal communication deficits in ASD in our study.

It was reported that response inhibition is consistently associated with the inferior and middle frontal gyrus, which may relate to the severity of core deficits in ASD (Voorhies et al., 2018). Functionally, during the inhibition condition task, the ASD initially engaged the right rather than the left frontal cortex (typically developing first recruited the left middle frontal gyrus) and had reduced recruitment of non-frontal regions, which is related to their difficulty in executing top-down control (Vara et al., 2014). In our study, increased rightward asymmetry of GM volume in the inferior and middle frontal gyrus was found in the RRB subgroup. Alterations in structural asymmetry may have functional consequences. Our results may explain the first recruitment of the right frontal cortex in ASD. The increased structural rightward asymmetry may be the foundation of long-range functional hypoconnectivity and local hyperconnectivity in the frontal cortex, which are the underlying deficits of RRB in ASD.

Unique GM asymmetry alterations in each subgroup underline that core-symptom-specific analyses of structural asymmetry are well motivated. For the reason of dividing the heterogeneous ASD subjects into symptom-related subgroups would increase the specificity and accuracy of neuroimaging findings.

### 4.3. Brain-behavior relationships

By multiple regression analysis, we found that the AIs in Cluster A4 (thalamus) were negatively associated with social scores measured by ADOS. The reduced social score (i.e., lower symptom severity) provides preliminary proof that increased rightward asymmetry in Cluster A4 might have a compensatory effect in regulating social deficits. Although ASD patients have inactive responses to social stimuli, in some cases this low interaction may be improved by early intervention (Cohen et al., 2018). The data here suggest that atypical rightward asymmetry of the thalamus may indeed be the anatomic basis of social interaction deficits in ASD, and timely and effective interventions may exert their effect through recombination and functional balance of social-cognitive brain networks (Cohen et al., 2018), including the thalamus. This effect may provide novel ideas and new targets for the future treatment of ASD. However, more reproducible studies are needed to confirm this relationship.

### 4.4. Common GM asymmetry patterns in the general ASD group and ASD subgroups

The general ASD group and three subgroups showed consistent changes in the middle temporal and precuneus gyrus. Both brain regions overlap with the default mode network (DMN), which may be the cocircuit responsible for three core symptom domains. In the DSM-5, only two domains are included, achieved by merging the first two domains indicated in the DSM-IV-TR (social interaction and communication domains) into the social communication domain (Shulman et al., 2020). The model indicates convergence of social interaction and communication deficits, but not with RRB. The potential effect of the new criteria has received extensive attention, with some studies inspecting the sensitivity and specificity of the revision (Guthrie et al., 2013). However, no study has examined brain structural alterations related to the revised diagnostic domains. In our study, it should be noted that although there were overlapping brain regions between the SI subgroup and VA subgroup, there were even more differences in asymmetry alterations, including different brain regions and varying directions (i.e., the postcentral gyrus). Therefore, we believe that it is more reasonable to divide general ASD patients into three core-symptom-related subgroups in brain structural research.

### 4.5. The effect of age

The results of the multiple regression analyses between AIs and age suggested that GM asymmetry may not be constant in minors. In TD controls, this manifested as increased rightward asymmetry from age 7 to 18 in Cluster A2. In patients with ASD, the abnormal GM asymmetry changed with age: Cluster V1 had more leftward asymmetry, and Cluster A5 had more rightward asymmetry. In corresponding TD controls, both clusters showed reverse asymmetry. This demonstrates that degrees of abnormality in Cluster V1 and Cluster A5 were increased. Whether these GM asymmetry alterations are caused by genetics, the environment, or both is unknown. Prior studies have shown that there are structural asymmetries present even before birth (Hepper et al., 1991; McCartney and Hepper, 1999). Autopsy histological studies have indicated that genetic factors contribute to brain lateralization (Karlebach and Francks, 2015). Our observations further illustrate the importance of early intervention for ASD. This is a key field for future study and warrants further longitudinal analysis.

### 4.6. Limitations

We examined the relationship between abnormal GM asymmetry and age but did not conduct longitudinal comparisons between the age effect and GM asymmetry due to the limitation of the sample size. To investigate the longitudinal change in GM asymmetry in each subgroup, it is necessary to employ the current grouping pattern and explore the effect of age on GM asymmetry using a higher-power approach with longitudinal tracking and a larger number of participants. Data in our study were screened from the ABIDE I database and collected from eight sites around the world, which resulted in additional variabilities, including differences in scanning equipment and parameters, clinical behavioral evaluation, and participant selection. However, the bias of the site was minimized by using this effect as a covariate when conducting statistical analyses.

## 5. Conclusion

To identify the neural substrates underlying different core symptom domains, we explored the core-symptom-related alterations in GM asymmetry in ASD. We found unique GM asymmetry changes in three ASD subgroups and some specific alterations across subgroups were greatly overshadowed in the general ASD group. These findings emphasize the role of core symptoms in exploring ASD-related neuroimaging alterations and provide a clear characterization of core-symptom-related differences in neurobiology. Furthermore, abnormal GM asymmetries increase in degree with age, illustrating the importance of early intervention and treatment. In addition, the compensatory effect of Cluster A4 in regulating social deficits may provide novel ideas and new targets for the future treatment of ASD. In conclusion, our results further support the theory that each core symptom domain of ASD may have independent etiological and neurobiological underpinnings, which is crucial to the future diagnosis and treatment of ASD.

## Data availability statement

The datasets presented in this study can be found in online repositories. The names of the repository/repositories and accession number(s) can be found in the article/Supplementary material.

## Ethics statement

Ethical review and approval were not required for the study on human participants in accordance with the local legislation and institutional requirements. Written informed consent from the participant’s legal guardian/next of kin was not required to participate in this study in accordance with the national legislation and institutional requirements.

## Author contributions

CL and MN conceived and designed the experiments. CL, MN, WC, XL, TL, YC, CZ, and XW analyzed the data. CL and MN wrote the manuscript. All authors contributed to the article and approved the submitted version.

## Footnotes

1
http://dbm.neuro.uni-jena.de/vbm8

2
http://www.fil.ion.ucl.ac.uk/spm

3
http://dbm.neuro.uni-jena.de/vbm8/TPM_symmetric.nii

4
https://www.nitrc.org/frs/?group_id=152

5
http://rfmri.org/dpabi

## Supplementary material

Supplementary Figure 1 Differences in GM asymmetry in between-group comparisons based on ADOS. **(A)** SI_2_ vs. TD: 3 clusters with significant differences between SI_2_ and TD controls. **(B)** VA_2_ vs. TD: 3 clusters with significant differences between VA_2_ and TD controls. The red color indicates SI_2_/VA_2_ with more rightward asymmetry, and the blue color indicates more leftward asymmetry. The results were corrected for multiple comparisons using the Gaussian random field procedure with the voxel level *P*-value < 0.005 and the cluster level of *P* < 0.05. GM, gray matter; ADOS, Autism Diagnostic Observation Schedule; SI_2_, subgroup dominated by social interaction deficits based on ADOS; VA_2_, subgroup dominated by verbal communication abnormalities based on ADOS.
