# Supplementary material for: Gray matter asymmetry atypical patterns in subgrouping minors with autism based on core symptoms
Source: Front Neurosci. 2023 Jan 25;16:1077908. doi: 10.3389/fnins.2022.1077908 (PMC9905125; doi:10.3389/fnins.2022.1077908)
Supplement: Supplementary file 1 [file Table_1.docx]

**Supplementary Table 1.** Participant demographics

|  | Variables | | USM | NYU | UCLA | PITT | KKI | SDSU | OHUS | Total |
| --- | --- | --- | --- | --- | --- | --- | --- | --- | --- | --- |
| VA_2_and TD | Subjects (N) | VA_2_ (TD) | 10(26) | 9 (29) |  |  |  |  |  | 19(55) |
|  | Age (years) | VA_2_ | 15.92±2.07 | 10.48 ± 4.05 |  |  |  |  |  | 13.24±4.15 |
|  |  | TD | 15.7±1.59 | 9.33±1.42 |  |  |  |  |  | 12.35±3.54 |
|  | Gender M(F) | VA_2_ | 10(0) | 7(2) |  |  |  |  |  | 17(2) |
|  |  | TD | 25(1) | 24(5) |  |  |  |  |  | 49(6) |
|  | Statistics | Age | *t*=0.32  *p*=0.75 | *t*=1.33  *p*=0.42 |  |  |  |  |  | *t*=1.01  *p*=0.32 |
|  |  | Gender | *Χ^2^* = 0.25  *p* = 0.61 | *Χ^2^* = 0.02  *p* = 0.88 |  |  |  |  |  | *Χ^2^* = 0.15  *p* = 0.70 |
| SI_2_ and TD | Subjects (N) | SI_2_ (TD) | 9(36) | 44(58) | 53(47) | 9(14) | 16(30) | 8(17) | 9(23) | 148(225) |
|  | Age (years) | SI_2_ | 16.35±1.57 | 11.58±2.35 | 12.87±2.33 | 14.41±2.20 | 10.08±1.54 | 14.19±1.77 | 12.01±2.08 | 12.51±2.61 |
|  |  | TD | 16.13±1.44 | 11.28±0.32 | 12.95±1.85 | 13.81±1.96 | 9.91±1.02 | 13.56±1.64 | 11.39±2.29 | 12.56±2.69 |
|  | Gender M(F) | SI_2_ | 9(0) | 40(4) | 46(7) | 7(2) | 14(2) | 7(1) | 9(0) | 132(16) |
|  |  | TD | 33(3) | 47(11) | 41(6) | 12(2) | 22(8) | 14(3) | 23(0) | 192(33) |
|  | Statistics | Age | *t*=0.40  *p*=0.69 | *t*=0.65  *p*=0.52 | *t*=-0.19  *p*=0.85 | *t*=0.69  *p*=0.50 | *t*=0.46  *p*=0.65 | *t*=0.86  *p*=0.40 | *t*=0.71  *p*=0.49 | *t*=-0.19  *p*=0.85 |
|  |  | Gender | *Χ^2^* = 0.02  *p* = 0.88 | *Χ^2^* =2.63  *p* = 0.11 | *Χ^2^* = 0.004  *p* = 0.95 | *Χ^2^* = 0.01  *p* = 0.94 | *Χ^2^* = 0.54  *p* = 0.46 | *Χ^2^* = 0.07  *p* = 0.80 |  | *Χ^2^* = 1.16  *p* = 0.28 |

**Abbreviations:** SI_2_, subgroup dominated by social interaction deficits measured by ADOS; VA_2_, subgroup dominated by communication abnormalities measured by ADOS; KKI, Kennedy Krieger Institute; NYU, New York University Langone Medical Center; UCLA, University of California-Los Angeles; PITT, University of Pittsburgh School of Medicine; OHSU, Oregon Health and Science University; SDSU, San Diego State University; USM, University of Utah

School of Medicine.
