# Supplementary material for: Gray matter asymmetry atypical patterns in subgrouping minors with autism based on core symptoms
Source: Front Neurosci. 2023 Jan 25;16:1077908. doi: 10.3389/fnins.2022.1077908 (PMC9905125; doi:10.3389/fnins.2022.1077908)
Supplement: Supplementary file 2 [file Table_2.docx]

**Supplementary Table 2** Coordinates of clusters with significant differences in between-group comparisons.

|  | Cluster | Location | MNI coordinates | | | voxels | t-value |
| --- | --- | --- | --- | --- | --- | --- | --- |
|  |  |  | x | y | z |  |  |
| SI_2_ vs TD | S1 | Middle Temporal Gyrus | 66 | -30 | -25.5 | 374 | -3.466 |
|  | S2 | Postcentral/Precentral Gyrus | 57 | -25.5 | 60 | 73/40 | 2.921 |
|  | S3 | Insula | 42 | -10.5 | 21 | 70 | 3.358 |
| VA_2_ vs TD | V1 | Superior Temporal/Middle Temporal Gyrus | 43.5 | -25.5 | -1.5 | 39/32 | -2.882 |
|  | V2 | ParaHippocampal | 10.5 | 1 | -24 | 41 | -2.812 |
|  | V3 | Precuneus | 3 | -66 | 24 | 22 | -2.635 |

**Abbreviations:** SI_2_, subgroup dominated by social interaction deficits measured by ADOS; VA_2_, subgroup dominated by verbal communication abnormalities measured by ADOS.
