# Supplementary figures and images for: Gray matter asymmetry atypical patterns in subgrouping minors with autism based on core symptoms
Source: Front Neurosci. 2023 Jan 25;16:1077908. doi: 10.3389/fnins.2022.1077908 (PMC9905125; doi:10.3389/fnins.2022.1077908)

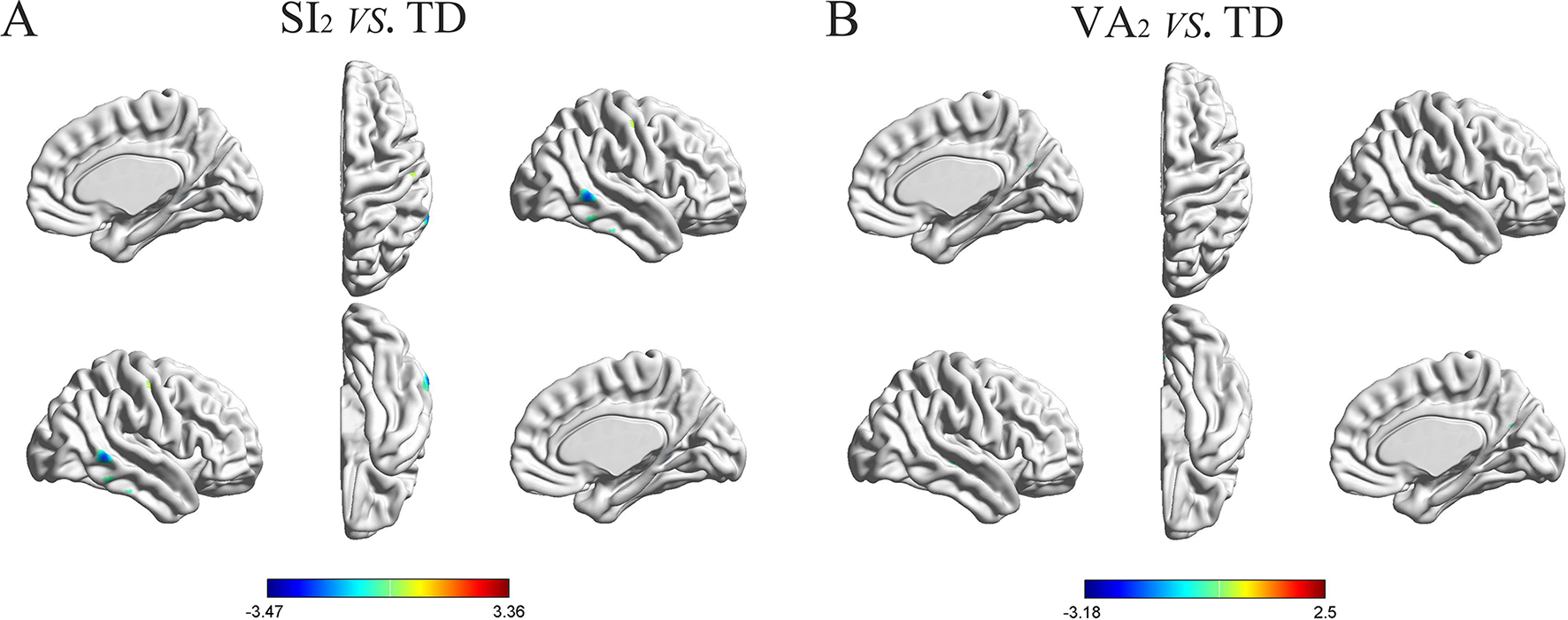

Supplement: Supplementary Figure 1 — Differences in GM asymmetry in between-group comparisons based on ADOS. (A) SI2 vs. TD: 3 clusters with significant differences between SI2 and TD controls. (B) VA2 vs. TD: 3 clusters with significant differences between VA2 and TD controls. The red color indicates SI2/VA2 with more rightward asymmetry, and the blue color indicates more leftward asymmetry. The results were corrected for multiple comparisons using the Gaussian random field procedure with the voxel level P-value < 0.005 and the cluster level of P < 0.05. GM, gray matter; ADOS, Autism Diagnostic Observation Schedule; SI2, subgroup dominated by social interaction deficits based on ADOS; VA2, subgroup dominated by verbal communication abnormalities based on ADOS. [file Image_1.TIFF]
